# Cortical GABA in migraine with aura -an ultrashort echo magnetic resonance spectroscopy study

**DOI:** 10.1186/s10194-019-1059-z

**Published:** 2019-12-03

**Authors:** Tobias G. Stærmose, Marie K. Knudsen, Helge Kasch, Jakob U. Blicher

**Affiliations:** 1 Center of Functionally Integrative Neuroscience, Institute of Clinical Medicine, Aarhus University Hospital, Aarhus, Denmark; 2 Spinal Cord Injury Center of Western Denmark, Department of Neurology Viborg Regional Hospital, Viborg, Denmark; 3 Department of Clinical Medicine, Aarhus University, Aarhus, Denmark

**Keywords:** Magnetic resonance spectroscopy, MRS, Migraine, Migraine with aura, MWA, GABA

## Abstract

**Objective:**

The aim of this cross-sectional study was to investigate the cortical metabolite concentrations in patients suffering from migraine with aura (MWA). We hypothesized that occipital γ-aminobutyric acid (GABA) levels are lower in MWA patients.

**Background:**

Recent studies have indicated that a disturbance in the inhibitory GABA is involved in triggering the migraine aura. We aimed to explore this using a novel magnetic resonance spectroscopy sequence.

**Methods:**

Using spin echo full intensity acquired localized spectroscopy on a Siemens 3 Tesla magnetic resonance scanner, we obtained occipital and parietal metabolite concentrations in 14 patients suffering from migraine with aura and a group of 16 matched healthy subjects.

All scans were performed at Aarhus University Hospital, at the Center for Functionally Integrative Neuroscience (CFIN).

**Results:**

No difference was found in GABA/(Total creatine) levels in either the occipital cortex (*p* = 0.744) or in the somatosensory cortex (*p* = 0.305).

**Conclusion:**

These findings indicate that cortical GABA levels are normal in patients suffering from relatively few migraine attacks. Previous studies have reported that cortical GABA in patients with more frequent migraines is reduced; further investigation of the inhibitory system in migraine patients is warranted to determine the underlying mechanisms.

## Introduction

Migraine is an episodic type of primary headache affecting a large proportion of the population with a female preponderance. Approximately 20% of all migraine patients experiences auras, often of the visual type [1]. Cortical spreading depression (CSD) is thought to be the physiological mechanism underlying migraine aura; hence, studies using both magnetic resonance spectroscopy (MRS) and visually evoked potentials indicate an abnormal inhibition-excitation balance [2–6]. An unbalanced inhibition-excitation system of the brain could be driven by a γ-aminobutyric acid (GABA) – Glutamate disturbance (see [7] for a recent review on GABA and migraine). If the GABA concentrations are found to be altered in migraine patients, this could lead to new preventative approaches in migraine treatment.

Previous MRS studies have investigated change of cortical GABA in migraine patients [2–5]. Studies were underpowered and inconclusive, MRS methods differed, and study population included a mixture of migraine patients with and without aura with large variability in headache frequency [8]. Based on prior studies by Bridge et al. [2] and Bigal et al. [5], it is possible that occipital GABA levels are reduced in frequent migraine. However, these findings have not yet been replicated. Furthermore, it is unknown whether GABA and/or glutamate levels are changed in migraine patients due to the migraine attacks, and it is also unknown if low GABA levels are part of the underlying mechanism triggering the migraine aura. Previous studies were not designed to examine if the diminished levels of GABA were due to migraine prophylactic medication.

The present study recruited mildly affected migraine patients with episodic aura of low frequency. It was hypothesized that occipital GABA levels were decreased in migraine patients as compared with healthy subjects. In order to replicate prior findings by Bridge et al. [2] and Bigal et al. [5], our primary focus was on the occipital cortex. Secondarily, the somatosensory (parietal) cortex was investigated to clarify whether any differences in the occipital cortex would generalize to other sensory areas.

The primary outcome for this study was difference in GABA/Total creatinine levels between patients with MWA and healthy matched subjects measured using Proton (H^+^) Magnetic resonance spectroscopy. Furthermore, the secondary outcome measures included comparison of total glutamate/glutamine (Glx) ratioed to total creatinine as well as additional metabolites in MWA patients and healthy controls, provided using the SPECIAL spectroscopy method.

## Methods

This cross-sectional imaging study was approved by the Central Denmark Region Research Ethics Committee (case number: 1–10–72-326-15) prior to patient recruitment. All participants gave written and verbal informed consent. All patients were scanned between September 2015 and June 2016 at Aarhus University Hospital, at the Center for functionally integrative neuroscience (CFIN), using a Siemens Trio 3 T scanner dedicated for research.

Participants were recruited using posters that were on public display in Aarhus University and at Aarhus University Hospital. Firstly, participants were questioned using a semi structured questionnaire, and then secondly to establish a migraine diagnosis, a trained research assistant (TB) and a specialist neurologist (HK) screened all potential patients before study enrolment. Inclusion criteria were: Migraineurs should fulfil criteria for episodic migraine with aura and with headache (diagnosis group IHS ICHD-3 1.2.1.1 https://www.ichd-3.org/1-migraine/), 4–20 attacks during the past year, age 18 to 50 years, no other significant illness and no medication known to affect neuronal excitability other than migraine treatment.

Patients was screened by the criteria that during the last 1 years at least 4 headache attacks with:

Typical aura with signs of vision disturbances or somatosensory disturbances eventually mild motor aura (speak difficulties, clumsiness) before or during headache, at least 4 headaches with aura episodes identified with full remission within hours-1 week.

Duration of each headache attack was reported from 4 h to 1–2 days if headache was untreated.

Typically, moderate intense headache, predominantly unilateral or eventually bilateral origin of headache, typically headache with pulsating/throbbing character. Other signs like nausea, vomiting, phono- and/or photophobia during headaches were registered, worsening during activity and improvement during rest. Participants were excluded if they reported any other neurological disorder.

Scans were performed at least 7 days after last migraine attack.

Control subjects were included in a similar fashion using a semi structured questionnaire and screening process. Inclusion criteria were: Healthy men or women, age 18–50. Exclusion criteria were any prior migraine or other headache diagnosis, any significant psychiatric diagnosis or any current neuro modulating medication. We did not collect information on any family history of migraine from the healthy subjects.

All scans were performed during the day time, from 8.00 AM to 4.15 PM, scheduled scans were performed independently of group affiliation.

### Magnetic resonance imaging and spectroscopy

The measurements were done using the spin echo full intensity acquired localized spectroscopy (SPECIAL) magnetic resonance method performed on a 3 T Magnetom Trio System (Siemens, Erlangen, Germany) with a body coil transmitter and a 32-channel head-coil. Each session consisted of a T1-weighted MPRAGE (TR/TE = 2420/4.6 ms, 1 mm isotropic resolution) structural scan for voxel positioning and segmentation.

**Fig. 1:**
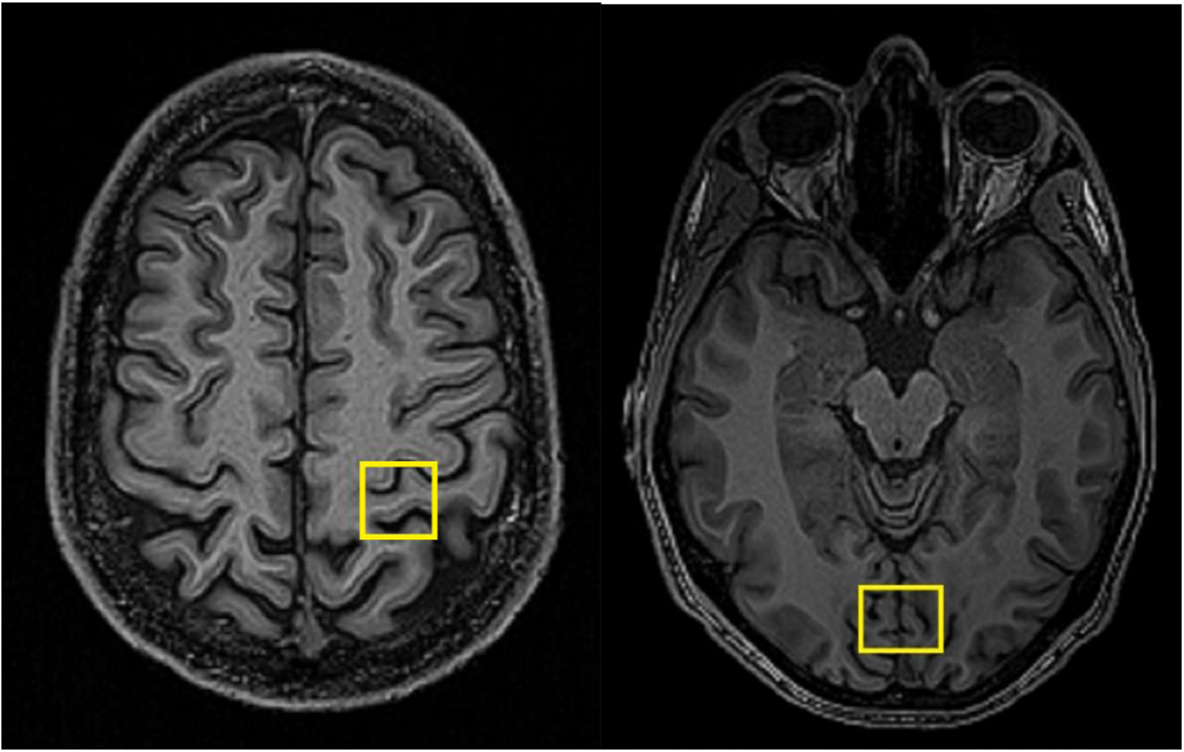
Example SPECIAL MRS voxel placement in the primary somatosensory cortex (left) and the occipital cortex (right)

The Proton (H^+^) Magnetic Resonance spectroscopy (MRS) was performed using spin echo full intensity acquired localized spectroscopy (SPECIAL) [9, 10] TR/TE 4000/8.50 ms with a 25x20x20 mm voxel placed in the occipital cortex (128 averages, scan time 8 min 48 s) and a 20x20x20 mm voxel placed in the primary somatosensory cortex of the dominant hemisphere (154 averages, scan time 10 min 58 s). See Fig. 1 for anatomical voxel placement.

For both voxels, an additional SPECIAL scan was performed to obtain a water unsuppressed spectrum with eight averages, used for eddy-current correction in the spectral analysis. A manual shim was performed using the fastest map [11, 12] (TR/TE 2000/44 ms, full 6-bar fit, 12 s scan time).

### Data analysis

The raw spectroscopy data was pre-processed in MATLAB (2015b, The MathWorks Inc., Natick, MA, 2015) using the FID-A [13] script to correct for bad averages, motion, frequency drift and to apply phase correction at zero- and first order to create a final averaged spectrum.

LCModel (v 6.3, Provencher, 1993) was used to fit the data and to quantify metabolites.

For every subject the T1 images were imported in Statistical Parametric Mapping 12 (www.fil.ion.ucl.ac.uk) running in MATLAB and segmented for grey matter (GM), white matter (WM) and Cerebrospinal fluid (CSF) content in the two voxel positions.

**Fig. 2:**
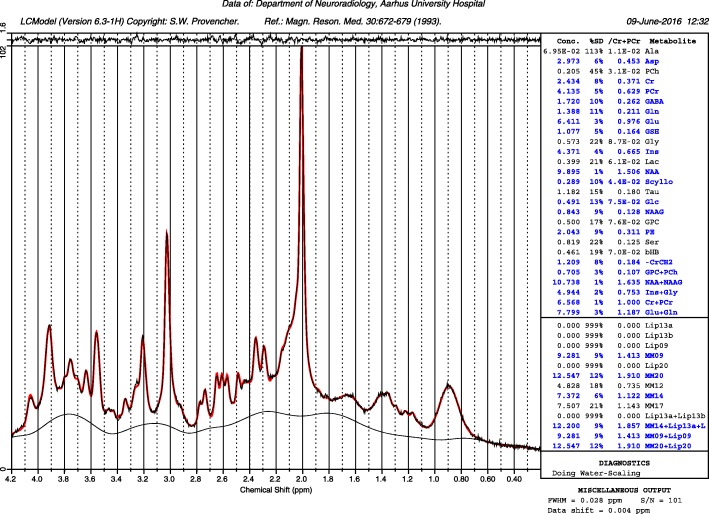
Top: Example spectrum from a representative migraine patient from the occipital voxel position. The red line is the LCModel fit of the data from this patient, the thin black line with the same shape as the red fit is the raw data. The baseline is the thin black line lowest in the spectrum. At the top of the plot is the residuals of the fitted raw data to the model, i.e. the raw data minus the fitted data. Quality data: for this spectrum: S/N 101 and a delta shift of 0.004 ppm, FWHM = 0.028 ppm. Bottom: The GABA fit from the same spectra

**Fig. 3:**
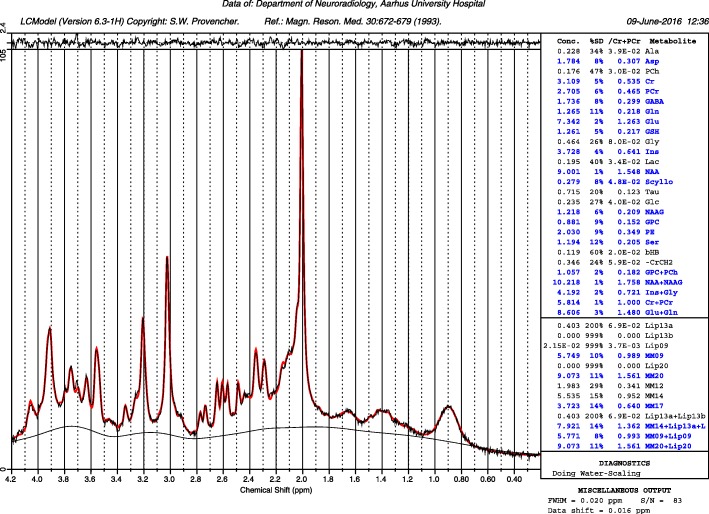
Top: Example spectrum from a representative migraine patient from the somatosensory voxel position. The red line is the LCModel fit of the data from this patient, the thin black line with the same shape as the red fit is the raw data. The baseline is the thin black line lowest in the spectrum. At the top of the plot is the residuals of the fitted raw data to the model, i.e. the raw data minus the fitted data. Quality data: for this spectrum: S/N 83 and a delta shift of 0.016 ppm, FWHM = 0.020 ppm. Bottom: The GABA fit from the same spectra

Several parameters were used as quality assessments of each of the spectra: Cramér–Rao lower bound (CRLB) above 20% for the individual metabolites, spectral line widths greater than 8 Hz or Signal-to-noise ratio (SNR) lower than 40 were removed from further analysis. All subjects included in the final analysis had spectra from both voxels that had quality parameters fulfilling the requirements. See Figs. 1, 2 and 3 for example spectra and GABA fit. Statistical analysis using two-tailed t-test was performed comparing MWA patients to healthy subjects using JASP (v. 0.11.1, 2019, https://jasp-stats.org) which was also use for Pearson correlation analysis. GM correction calculations was done using Microsoft Excel (Microsoft Office 365 Pro Plus 64-bit, 2019, v. 16.0.411328.20438).

The researcher who performed the analysis was blinded to the type of subject and voxel position.

### Statistics

In the previous study by Bridge et al. [2] a significant difference was found comparing 11 migraineurs to healthy subjects. Data allowing for a complete pre-study power calculation were not available and thus we assumed that a minimum of 15 patients would have significant power to show alterations in GABA concentrations in migraine patients. Results are reported as mean and standard deviation (SD). The criteria for statistical significance was α *p* < 0.05. We used a Bonferroni correction on all secondary metabolites (*p*-value/number of tests).

## Results

Sixteen migraine patients were included, one female patient was unable to complete the scan due to claustrophobia. One migraine patient was in migraine prophylactic treatment with lamotrigine and was excluded in the final analysis. Seven patients were prescribed triptans for migraine attacks. Seventeen age- and gender-matched healthy subjects (10 women, 7 men) were included for comparison. One control data set was lost due to technical problems. The final analysis concluded a sample consisting of 14 patients (9 women, mean age of 23 (range 19–34)) and 16 healthy subjects (9 women, mean age of 23 (range 19–33)). The mean number of migraine attacks were 11.04 per year (Range 4–25 attacks/year).

### Magnetic resonance spectroscopy findings

Data quality was good, and the SPECIAL method provided data of good quality on GABA metabolites as well as on several other metabolites being secondary outcome parameters. All spectra in the final analysis passed our quality criteria.

#### Occipital cortex

In the occipital lobe no difference in GABA/Cr + PCr (Total Creatinine) levels was observed between the migraine patients (mean 0.305 ± 0.054) and the healthy subjects (mean 0.298 ± 0.055) *P* = 0.744. In order to examine if the results were confounded by a difference in total creatinine, we compared GABA/NAA + NAAG(*N*-acetylaspartate + N-acetylaspartylglutamate) ratios and found no significant difference between migraineurs (mean 0.165 ± 0.033) and healthy subjects (mean 0.173 ± 0.024) *P* = 0.441. Comparing water corrected GABA concentrations showed no differences between patients (mean 1.780 ± 0.271) and healthy subjects (mean 1.891 ± 0.284) *P* = 0.283. We also tested if total creatinine (Cr + PCr) differed, but the levels in migraineurs (mean 6.009 ± 0.446) and in healthy subjects (mean 6.241 ± 0.369) were not significantly different *P* = 0.131. Finally, we corrected the individual voxels for grey matter (GM) content using the formula: (GABA/Cr + PCr)/(GM/(GM + WH + CSF)). No difference between patients (mean 0.437 ± 0.082) and healthy subjects (mean 0.446 ± 0.082) was observed *P* = 0.774, when testing GM corrected GABA/Cr + PCr ratios.

#### Somatosensory cortex

In the Somatosensory cortex GABA/Cr + PCr levels were similar between patients (mean 0.286 ± 0.045) and healthy subjects (mean 0.270 ± 0.038), *P* = 0.305. Water corrected GABA showed no difference between patients (mean 1.638 ± 0.225) and healthy subjects (mean 1.597 ± 1.83) *P* = 0.592), neither did GABA/NAA + NAAG levels (*P* = 0.979), and Grey matter corrected GABA/Cr + PCr values (*P* = 0.167).

#### Other metabolites

As the SPECIAL technique allows for analysis of multiple metabolites in the same voxel, a secondary aim of the study was to test for any differences in these metabolites (Tables 1, 2 and 3).

**Table 1:** Quality of spectral data

Quality parameters	Group	Occipital		Somatosensory	
		Mean	SD	Mean	SD
SNR	Control	89.063	6.777	68.000	6.693
	MWA	89.429	12.482	62.429	9.010
FWHM (ppm)	Control	0.033	0.005	0.025	0.004
	MWA	0.051	0.066	0.026	0.004
Delta Shift	Control	0.012	0.012	0.012	0.013
	MWA	0.006	0.014	0.004	0.011

Student’s t-test (*p*-values)

**Table 2:** CRLB data

CRLB Metabolite	Group	Occipital	Somatosensory
		Mean	Mean
GABA	Control	9.750	10.813
	MWA	10.571	11.357
Glx	Control	3.625	4.249
	MWA	3.714	4.071
NAA + NAAG	Control	1.063	1.000
	MWA	1.143	1.071
Asp	Control	6.313	9.188
	MWA	6.357	9.929
Ins	Control	4.063	3.875
	MWA	4.071	4.214
Cr	Control	9.313	8.688
	MWA	9.357	9.071
PCr	Control	6.063	6.938
	MWA	6.071	7.143

**Table 3:** All values are presented as mean ratios, Two-sample t-tests

Group Descriptives		Occipital			Somatosensory		
Metabolite	Group	*p*	Mean	SD	*p*	Mean	SD
GABA (water)	Control	0.283	1.891	0.284	0.592	1.597	0.183
	MWA		1.780	0.271		1.638	0.225
GABA/Cr + PCr	Control	0.744	0.305	0.054	0.305	0.270	0.038
	MWA		0.298	0.055		0.286	0.045
GABA/NAA + NAAG	Control	0.441	0.173	0.024	0.979	0.169	0.017
	MWA		0.165	0.033		0.169	0.020
Glx (water)	Control	0.107	7.991	0.704	0.438	7.362	0.548
	MWA		7.646	0.345		7.536	0.662
Glx/Cr + PCr	Control	0.851	1.271	0.094	0.111	1.244	0.105
	MWA		1.278	0.097		1.314	0.129
Asp (water)	Control	0.710	2.675	0.339	0.790	1.903	0.225
	MWA		2.718	0.290		1.923	0.175
Asp/Cr + PCr	Control	0.116	0.429	0.045	0.206	0.321	0.032
	MWA		0.452	0.032		0.335	0.028
Cr + PCr (water)	Control	0.131	6.241	0.369	0.134	5.937	0.388
	MWA		6.009	0.446		5.747	0.264
Ins (Water)	Control	0.220	4.349	0.558	0.065	4.588	0.389
	MWA		4.113	0.459		4.307	0.413
Ins/Cr + PCr	Control	0.297	0.745	0.200	0.440	0.774	0.064
	MWA		0.685	0.071		0.752	0.092
NAA + NAAG (water)	Control	0.828	10.946	0.725	0.158	9.460	0.399
	MWA		10.888	0.738		9.712	0.550
NAA + NAAG/Cr + PCr	Control	0.012*	1.742	0.090	0.015**	1.598	0.089
	MWA		1.834	0.098		1.693	0.111

Student’s t-test (*p*-values)

* *P* = 0.012, ** *P* = 0.015 comparing migraine patients to healthy subjects, both considered non-significant after Bonferroni correction

Several other metabolites being secondary outcome parameters were examined, no significant differences were detected between MwA and healthy subjects. NAA + NAAG/Cr + PCr tests showed *p*-values below 0.05 but were considered non-significant after Bonferroni correction. To further investigate we then also tested NAA + NAAG referenced to water and total Creatinine referenced to water, no difference was found.

We tested whether the number of migraine attacks per year correlated to the level of GABA (water referenced), GABA/Cr + PCr, Glx (water referenced) and Glx/Cr + PCr, no correlation was found (all *p*-values above 0.05).

## Discussion

The present case-control study population with 14 mildly affected patients suffering from migraine with aura (MWA) (diagnosis group IHS ICHD-3 1.2.1.1 Migraine typical aura with headache (episodic) https://www.ichd-3.org/1-migraine/) was not able to replicate prior findings of low GABA levels in occipital cortex using functional MRI [2].

A disturbance in cortical excitatory/inhibitory balance has been suggested to contribute to the initiation of cortical spreading depression and thereby eliciting aura in MWA patients. Moreover, disturbed perception of visual stimuli has been documented in MWA patients [14] even outside of migraine attacks, which suggests an underlying disturbance in cortical function in migraineurs. To our knowledge three prior studies have investigated GABA levels using MRS in migraine patients, reporting high [3], low [2] or normal [5] GABA. The diverging results are likely due to differences in voxel placement (occipital vs parietal), MR-sequences used (MEGA-PRESS, SPECIAL), patients included (with or without aura) and disease severity. The prior studies by Bigal et al. [5] and Bridge et al. [2] both investigate occipital GABA in MWA patients (although the Bigal study included a mixed group of patients) and are thus comparable to the current study. In the study by Bigal et al. no difference was found between patients and healthy subjects, however a secondary analysis showed that GABA was lower in the patients with one or more severe migraine attacks within the last month. The recent study by Bridge et al. reported low occipital GABA levels in a cohort of MWA patients with a high frequency of migraine attacks (average 5 per month) and with several patients on migraine prophylactic treatment [2]. Taken together, the results from the two studies indicate that GABA levels are low in patients with more severe migraine. The current study investigated a group of more mildly affected patients (average of 11.04 attacks per year) undergoing MRI more than 7 days after their last registered migraine attack, making this sample very different from the patients examined by Bridge et al. In the present study GABA levels were unchanged in migraine patients with less severe affection, however the current study did not contribute with additional information regarding GABA levels in severe migraine headaches.

Using the SPECIAL MRS sequence, we also looked in to several other metabolites, such as Glutamate/Glutamine (Glx) and NAA (NAA+ NAAG). NAA levels have previously been found to be lower in migraine patients [8], we found no significant difference. As we work with the hypothesis that the migraine aura is caused by an imbalance in the excitatory-inhibitory system, glutamate could be of interest since it is one of the primary excitatory neurotransmitters in the brain, here we found no difference.

One of the major limitations of the current study is the lack of precise phenotyping, as well as the low number of participants. The study size is of concern both in this and in several previous studies and should be addresses in future studies.

Future studies should also address whether low GABA levels in severe migraine headache are due to migraine aura, or are a consequence of migraine prophylactic treatment, although evidence shows that prophylactic neuromodulators in migraine seem to elevate GABA levels [15], or are simply lowered during the migraine attack and the days after, and thus a consequence of migraine headache rather than the cause. In future studies the fact that some patients only experience unilateral MwA and, some have changing laterality would possibly enable you to use patients as internal reference, using the affected side vs the unaffected side.

Several studies suggest an imbalance in the GABA-glutamate system in migraine, the current study in mildly affected MwA patients does not support this hypothesis.

More control over patient migraine prophylactic medication would also be of great interest in future studies, in the present study no patients was taking prophylactic medication. The current available studies, the present one included show that more specific studies are needed to elucidate the nature of the different levels reported in the present study and in previous findings and their correlation to migraine.

## Conclusion

We did not find significant difference in cerebral GABA levels between patients and healthy subjects, indicating that in relatively mild migraine with aura GABA levels in the occipital and parietal lobes are normal outside migraine attacks.

## Key findings

- Using the SPECIAL MRS sequence, we obtained GABA spectra of excellent data quality. GABA levels in both occipital and parietal cortex were similar in MWA patients and healthy subjects.
- Our results did not confirm prior reports of low occipital GABA in MWA patients. However, as the current study enrolled patients suffering from a low frequency of migraine attack, it is possible that GABA is low in MWA patients with more frequent attack, as has been suggested by prior studies.

## Data Availability

The raw data for this study (MRI and MRS files) have embedded personal data from the test subjects, there for we are not able to provide any of the data for public access in order to protect the test subjects and comply with the data protection law.

## Abbreviations

Asp: Aspartate
Cr + PCr: Total creatinine
CRLB: Cramér–Rao lower bound
CSD: Cortical spreading depression
CSF: Cerebrospinal fluid
GABA: γ-aminobutyric
GM: Grey matter
Ins: Inositol
MRS: Magnetic resonance spectroscopy
NAA: *N*-acetylaspartate
NAAG: N-Acetylaspartylglutamate
SNR: Signal-to-noise ratio
SPECIAL: Spin echo full intensity acquired localized spectroscopy
Total Glutamate, Glx: Glutamate + Glutamin
WM: White matter
